# Examining the role of patient-reported external factors and risk of relapse in anti-neutrophilic cytoplasmic autoantibody vasculitis

**DOI:** 10.3389/fneph.2024.1404451

**Published:** 2024-07-02

**Authors:** Mary M. Collie, Dhruti P. Chen, Yichun Hu, Lauren N. Blazek, Vimal K. Derebail, Eveline Y. Wu, Koyal Jain, Nicole Orzechowski, Caroline J. Poulton, Candace D. Henderson, Ronald J. Falk, Susan L. Hogan

**Affiliations:** ^1^ University of North Carolina (UNC) Kidney Center, Division of Nephrology and Hypertension, Department of Medicine, UNC School of Medicine, Chapel Hill, NC, United States; ^2^ Division of Pediatric Allergy, Immunology, and Rheumatology, Department of Pediatrics, University of North Carolina School of Medicine, Chapel Hill, NC, United States; ^3^ Division of Allergy, Immunology, and Rheumatology, Department of Medicine, University of North Carolina School of Medicine, Chapel Hill, NC, United States; ^4^ Department of Neurology, University of North Carolina School of Medicine, Chapel Hill, NC, United States

**Keywords:** ANCA vasculitis, survey study, psychological events, insect bites, infections, disease relapse

## Abstract

The role of stressors, insect bites, and infections on disease relapse of ANCA vasculitis has yet to be entirely explored, with limited retrospective studies focused on disease onset from small participant cohorts. Our study analyzes longitudinal survey data from 2011–2022 to evaluate this perspective from a large ANCA vasculitis cohort. We collected surveys every three to six months to obtain information on self-reported psychological stressors and significant life events, insect bites, and infections throughout clinical disease. We defined cohorts as those who relapsed (Relapse Cohort) and controls as those who did not relapse (Remission Cohort) during the study period. Survey responses were retrospectively reviewed during a 15-month timeframe prior to relapse or during 15 months of remission and categorized by type of stress event, insect bite, and infections at every available 3-month interval. There were no significant differences in stress and insect bites between the relapse and remission cohorts. Patients who relapsed reported more frequent upper respiratory infections and other infections, such as those affecting the skin and eyes, but there were no significant differences in the incidence of pulmonary or urinary infections compared to the remission cohort. There was a significant difference in reported upper respiratory infections 9 to 15 months prior to the relapse date, indicating a remote history of infections as a potentially significant physical stressor that may contribute to disease relapse. More frequent patient-reported infections, specifically upper respiratory infections, may contribute to patient vulnerability to relapse. Counseling and close monitoring of patients after infectious symptoms could aid in earlier detection of disease flares. Future studies are essential to further understand the importance of distal risk factors and how they impact relapse.

## Introduction

Anti-neutrophil cytoplasmic autoantibody (ANCA)-associated vasculitis is a relapsing and remitting autoimmune disease characterized by vessel inflammation in multiple organ systems (1–5). The immunopathology of ANCA vasculitis is in part driven by autoantibodies to two known autoantigens, myeloperoxidase (MPO) and proteinase 3 (PR3). Identifying psychological and environmental risk factors reported by patients throughout the disease course is essential to gaining a more comprehensive understanding of the relapsing and remitting patterns characteristic of ANCA vasculitis.

The biological response from stress in modulating immune function suggests a plausible link between stress and autoimmunity characterized by tissue inflammation and damage (6). Previous studies have shown that stress plays a role in various autoimmune diseases, including rheumatoid arthritis (RA), childhood arthritis, systemic lupus erythematosus, Graves’ disease, and Sjögren’s syndrome (6–8). In response to stress, cortisol released from activation of the hypothalamic-pituitary-adrenocortical axis regulates anti-inflammatory responses (9). Similarly, catecholamines released from the sympathetic-adrenal-medullary and autonomic nervous systems regulate cardiovascular, pulmonary, hepatic, skeletal, and immune systems (10). Persistent exposure to stressful events or continuous recall of stressful events prolongs endocrine response pathway activation, which is thought to cause long-term emotional, physiological, and behavioral changes. These changes may influence susceptibility to disease relapse and alter the long-term course of the disease (9, 10). Understanding the role of stress in autoimmune disease activity could have implications for disease management and treatment.

The interaction between environmental factors and autoimmune disease is not yet fully understood either. Environmental exposures such as insect bites or infections are thought to play a role in the dysregulation of inflammatory responses, leading to overproduction of autoantibodies in genetically predisposed individuals. Exposure to foreign antigens from infection or insect bites may induce autoimmunity through molecular mimicry, epitope spreading, or bystander activation (11, 12). Patient-reported psychological stressors, insect bites, and infection have not been studied previously for their potential association with relapsing ANCA vasculitis. Current literature is limited to retrospective evaluation of stress and coping strategies with surveys administered after disease activity, including disease onset or relapse (13–15).

We evaluated data from surveys of patients with ANCA vasculitis over a longitudinal period with patient-reported events throughout their disease course. The focus of this investigation was to identify self-reported psychological stressors including significant life events, insect bites, and infections during disease relapse and remission. Understanding the role of these exposures in ANCA vasculitis could have implications for disease monitoring, management, and treatment.

## Materials and methods

### Study participants

Patients with ANCA vasculitis were enrolled at UNC Health clinics and followed in the Glomerular Disease Collaborative Network (GDCN) longitudinal patient registry (16, 17). Patients were diagnosed according to Chapel Hill Consensus Conference criteria (18, 19). Patients were initially recruited from the GDCN registry and provided informed, written consent to participate in sub-study in accordance with the UNCCH Office of Human Research Ethics Institutional Review Board guidelines (IRB study 97–0523). A total of 162 eligible participants with an existing clinical diagnosis of ANCA vasculitis were enrolled. Secondary data analysis was performed under an ancillary study IRB (22–0598), eventually including 112 participants with clinically defined disease status and longitudinal survey follow-up. The 50 participants excluded did not have clinician confirmed relapse or remission dates and/or were lost to follow-up with no available surveys.

Disease activity was defined by the 2003 Birmingham Vasculitis Activity Score (BVAS) and confirmed through clinician chart review (20). Active disease (onset or relapse) was defined as BVAS >0 with clinical and/or laboratory evidence of disease and/or new or escalation of immunosuppressive therapy. Patients with a BVAS of 0 and no clinical or laboratory evidence of active disease within 3 months of the clinical follow-up date were considered in remission. Complete remission after initial diagnosis was required prior to relapse. ANCA serotypes were assessed by indirect immunofluorescence with positive cytoplasmic (C-ANCA) or perinuclear (P-ANCA) staining and antigen-specific PR3 and MPO enzyme-linked immunosorbent assays (ELISA) (20). MPO- and/or P-ANCA were classified together and PR3- and/or C-ANCA were classified together. Patients with only P-ANCA positivity prior to MPO availability were required to have a negative Antinuclear Antibody (21).

Detailed treatment regimens were not analyzed with patient surveys for this study but all patients received comparable treatment as the cohort is out of a single center. At our center, patients who have severe organ-threatening disease are treated with induction therapy, which consists of corticosteroids, cyclophosphamide (unless contraindicated or not tolerated), and/or rituximab, and 15 to 20% also receive plasmapheresis (22). This regimen is followed by maintenance dosing of rituximab for an additional 12–18 months, which is tailored to each patient in the context of other clinical factors (such as infections). Oral maintenance agents, including azathioprine and mycophenolate mofetil, are used in cases where rituximab may not be appropriate. Patients can come off all immunosuppressive therapy for periods of time during their disease course. End of immunosuppression is defined as: the date corticosteroids, azathioprine, mycophenolate mofetil, or oral cyclophosphamide are stopped, 30 days post-treatment of intravenous cyclophosphamide, or the date CD19+ B cells are detected in circulation or 1 year after the last dose if B-cell measures were not available for rituximab (22).

#### Determination of relapse and remission cohorts

Participants with at least 15 months of survey data prior to disease relapse during survey enrollment were defined as the relapse cohort. For the relapse cohort, the index date was the first relapse episode which occurred during the survey period. The remission cohort included participants with no relapse episodes, as determined by physician chart review, after the latest remission date before or on the survey enrollment date. Those in the remission cohort were required to remain in remission for a minimum of 15 months during the entirety of the survey collection period with chart review confirmed disease status. In order to compare exposures between cohorts, an index date was set at 15 months after starting remission for the remission cohort. Exposures during the 15 months of remission for the remission cohort were compared to the 15 months prior to relapse for the relapse cohort (**Supplementary Figure 3-1**).

### Survey collection and review

Surveys were collected at three-to-six-month intervals and administered in-person during clinic visits from July 2010 to May 2022. The survey questionnaire was composed by the study team for research done under the Program Project, ANCA Glomerulonephritis: From Molecules to Man (National Institutes of Health, P01DK05833, PI: RJF). The survey questions pertained to a broad range of patient-reported events and exposures in the last three months (see **Supplementary Material**). Patient-reported responses were recorded and retrospectively reviewed for this case-control study, comparing participants who relapsed to those who remained in remission following survey enrollment.

During survey collection, participants provided yes or no responses pertaining to infections and insect/tick bites with an extended request if answered yes for further details on the date and type of event. Insect bites were reported based on origin from tick, mosquito, unknown, or other. There were several questions on the survey requesting information on recent hospitalizations, new diagnoses, difficulties from disease burden, and notable life events that collected information on stressors within free text fields. Data from free text fields were systematically reviewed by one individual (MMC) and stress events were categorized as biological, environmental, cognitive, personal behavior, life situation, and other. Infections were categorized as upper respiratory infection, lower respiratory infection, urinary tract infection, or other infection. If a specific categorization for any response was not clear, the survey was reviewed and responses were categorized by committee (MMC, DC, KJ, SH) and a consensus was reached.

We reviewed the entire study cohort with survey responses and correlated clinical disease activity across survey duration to categorize participants into relapse and remission cohorts. Clinical disease activity was determined separately in clinic or in systematic review of medical records by a clinician (DPC, EYW, VKD and/or RJF) and a study team member (LNB, CEJ). A standardized 15-month timeline for all participants provided uniform cohort timeframes for comparison (**Supplementary Figure 3-1**).

### Statistical analysis

Descriptive statistics include numbers with percentage or median with interquartile range. Demographic characteristics and exposures of interest (stressors, insect bite, and infection) were compared between relapse and remission cohorts using Fisher’s exact test for categorical variables and Wilcoxon Two-Sample Test for continuous variables.

We collected records of stress, insect bites, and infection events from survey measurements. However, some survey measures were incomplete, resulting in missing event records. To address this, exclusion and inclusion criteria were used. Consequently, instead of counting the frequency of events, we calculated an average event occurrence in three months at –15, -12,…, -3 and 0 months to mitigate bias.

The average event rate was defined using the average infection event divided by total observation time for an infection type in three months. These rates over time from –15 to 0 months were compared between relapse and remission groups using Exact Cochran-Armitage Test. P-values less than 0.05 were considered statistically significant. All analyses were conducted with SAS (v.9.4, Cary, N.C.).

## Results

### Cohort characteristics

Survey data was analyzed from two groups as described in the Methods: those who experienced relapse (n=65 participants) and those in remission (n=47 participants). The relapse and remission cohorts were 55% and 47% female participants, respectively (p=0.44). The median age at the onset of disease was 52 years for the relapse group and 49 years for the remission group (p=0.56). The time from disease onset to enrollment in the survey was a median of 3 years for the relapse cohort and 1 year for the remission cohort. The cohort characteristics are shown in **Table 1**. The breakdown of serotypes within each cohort was as follows: 58% PR3-ANCA and 42% MPO-ANCA in the relapse group, and 44% PR3-ANCA and 56% MPO-ANCA in the remission group. Additionally, 1% of the relapse group and 9% of the remission group were dual positive for MPO and PR3-ANCA, or seronegative (p=0.17). **Table 2** outlines the available longitudinal survey data for each cohort. The relapse cohort (n=65) had a median follow-up of 8 years with an average of 19 surveys per participant. In comparison, the remission cohort (n=47) had a median follow-up of 3 years with an average of 7 surveys per participant.

**Table 1:** Cohort demographics.

Variables: n(%) or Median (IQR)	Relapse Cohort N=65	Remission Cohort N=47	P value *
**Sex**			0.44
**Female**	36(55%)	22(47%)	
**Male**	29(45%)	25(53%)	
**Age of Disease Onset**	52(31,60)	49(39, 63)	0.56
**Years from Diagnosis to Enrollment**	3(1,7)	1(0, 5)	0.02
**Years of Survey Follow-up**	8(5, 10)	3(1,5)	<0.0001
**ANCA**			0.17**
**PR3**	37(58%)	19(44%)	
**MPO**	27(42%)	24(56%)	

*P values were calculated by Fisher Exact test for categorical variables and Wilcoxon Two Sample Test for continuous variables.

**ANCA disease type also included n=5 dual positivity or negative type, not reported (1 Relapse, 4 Remission).

**Table 2:** Characterization of exposures for relapse and remission cohorts.

Variables: n(%) or Median (IQR)	Relapse Cohort N=65	Remission Cohort N=47	P value *
**Survey Number**	242	143	
**Surveys per Subject**	19(13,25)	7(3,10)	<0.0001
**Total External Exposures**	63(97%)	41(87%)	0.065
**Any Stressors**	58(98%)	36(77%)	0.12
**Number of Stressors**	2(1,3)	2(1,3)	0.41
**Insect Bites**	21(32%)	15(32%)	1.00
**Number of Insect Bites**	0(0,1)	0(0,1)	0.97
**Infections**	46(71%)	26(55%)	0.11
**Number of Infections**	1(0,2)	1(0,1)	0.03

*P values were calculated by Fisher Exact Test for categorical variables and Wilcoxon Two Sample Test for continuous variables.

All study participants received maintenance therapy during their clinical care during and/or outside of the observed survey period, with 88% receiving rituximab, 54% azathioprine, and 49% mycophenolate mofetil. Moreover, 58% of total participants stopped all immunosuppressive therapy at least once in their disease course.

In the relapse cohort, organ involvement at relapse was documented as 48% kidney, 42% upper respiratory, 25% joints, 12% lungs, 12% musculoskeletal, 9% neurological, and/or 8% dermatological. In the remission cohort, 70% of participants were in complete remission off therapy during the reviewed survey period, while 30% were in remission on therapy.

### Reported exposures

The frequency of external exposures reported at any time during the 15 months prior to relapse or the remission index date is shown in **Table 2**, along with the patient-reported incident frequency of each category of exposure (e.g., stress, insect bites, and infections). Overall, the frequency of any external exposure was not statistically significant between the relapse and remission cohorts (97% versus 87%, respectively, p=0.065). Stress events were also not significantly different between the relapse and remission cohorts (98% versus 77%, p=0.12), and the number of any stressors reported was similar, both with a median of 2 (p=0.41). Reported insect bites were also similar between the two groups (32% in each, p=1.00). Although the reported incidence of infections was not statistically significant between the relapse and remission cohorts (71% versus 55%, p=0.11), the median number of infections reported was statistically higher among the relapse cohort compared to the remission cohort (p=0.03). This finding led us to further explore specific infection types and rates of occurrence.

Of note, subgroup analyses limited to PR3 ANCA serotype and then to MPO ANCA serotype, did not reveal differences in frequency or counts of the exposures between the relapse and remission cohorts (**Supplementary Tables 2-1**, **2-2**).

### Reported infections

The average infection rate of upper respiratory, pulmonary, urinary, and other infections for relapse and relapse cohorts in 3-month intervals over 15 months were compared (**Table 3**). The relapse cohort consistently had higher rates of upper respiratory infections more than 6 months prior to the relapse as compared to this same time period prior to the remission index date (Exact Cochran-Armitage Test, P <0.0001, **Supplementary Figure 3-2A**). Reported pulmonary and urinary infections were lower compared to the rate of reported upper respiratory infections but were generally similar over time between the relapse and remission cohorts (Exact Cochran-Armitage Test, P-value= 0.08 and 0.10, **Supplementary Figure 3-2B, C**). “Other reported infections”, which was inclusive of those affecting the skin, eyes, oral cavity, gastrointestinal, or nervous system, were also significantly different between the relapse and remission cohorts (Exact Cochran-Armitage Test, P < 0.0001). The rate of other infections peaked at 15 and 6 months prior to the index date in the relapse cohort, in contrast to an increase in average cases from 15 to 3 months prior to remission index date (**Supplementary Figure 3-2D**). A subgroup analysis did not show serotype specific differences in the exposures (**Supplementary Tables 2-1**, **2-2**).

**Table 3:** The comparisons of the average rate of infection in infection types for relapse and remission patients***.

Infection Type (Disease status)	Interval (Months)						P value*
	-15	-12	-9	-6	-3	0	
**Upper Respiratory**							**<0.0001**
Relapse	30(16%)	45(23%)	42(22%)	34(18%)	10(5%)	32(17%)	
Remission	0(0%)	13(11%)	10(9%)	19(17%)	26(23%)	46(40%)	
**Pulmonary**							**0.08**
Relapse	4(24%)	5(29%)	0(0%)	3(18%)	0(0%)	5(29%)	
Remission	0(0%)	13(25%)	10(19%)	10(19%)	0(0%)	20(38%)	
**Urinary**							**0.10**
Relapse	9(39%)	5(22%)	9(39%)	0(0%)	0(0%)	0(0%)	
Remission	13(20%)	27(42%)	10(15%)	0(0%)	0(0%)	15(23%)	
Other**							**<0.0001**
Relapse	21(19%)	14(13%)	9(8%)	34(31%)	10(9%)	20(19%)	
Remission	0(0%)	13(14%)	10(11%)	10(11%)	34(37%)	25(27%)	

*P values were calculated using the Exact Cochran-Armitage Test.

**Other infections include those affecting the skin, eyes, oral cavity, gastrointestinal, or nervous system.

***The average infection rates were reported using per 100 persons with 100 measures within three months.

## Discussion

Prior to this study, the role of patient-reported psychological, environmental, and biological effects on the onset and progression of ANCA vasculitis had only been examined through limited retrospective studies focused on disease onset involving small participant cohorts. Our research marks a significant advancement as it is the first to employ survey data collection of exposures from a substantial cohort monitored over an extended period. By integrating longitudinal survey data obtained from individuals with ANCA vasculitis throughout their clinical journey, we gain invaluable insights into patient-reported experiences during both disease relapse and remission phases. We implemented rigorous criteria to delineate relapse and remission cohorts and index dates to ensure a robust comparison of exposures between these distinct disease states. In acknowledgment of cohort characteristics more predisposed to relapse (23), subgroup analysis for the PR3 and MPO-ANCA serotypes was carried out and did not suggest an influence on our findings, but there was limited statistical power. With meticulously defined relapse and remission groups followed longitudinally, we were able to discern patterns and correlations with greater clarity and precision.

Our study revealed a noteworthy pattern: patients who experienced relapse had reported upper respiratory infections between 9 to 15 months prior to their relapse, and these occurred more frequently than patients who remained in remission. This underscores the potential significance of remote past infections as a physical stressor contributing to disease relapse. We observed a significant increase in upper respiratory infections in the relapse cohort during the 15 to 9 months leading up to their relapse, compared to those in remission. Interestingly, other types of infections showed similar trends between the two cohorts during the 9 to 12 months preceding index dates. However, in the last 3 months before the relapse episode, both upper respiratory and other infections were less frequent in the relapse cohort compared to the remission group. Clinicians tend to focus on recent events and exposures when assessing patients; however, our findings suggest that past infectious exposures reported by patients may exert a lasting influence on the risk of relapse. Further research is warranted to validate these findings and to unravel mechanisms through which infections, even those occurring more than nine months prior, influence the immunological responses driving disease relapse. We do not have granular data regarding B cell repopulation to correlate immunological status with survey results. A specifically designed prospective study would need to be carried out to combine objective data from therapy effects (including B cells) and subjective, patient reported events to develop these measures into a predictive model for clinical use.

A notable strength of this study lies in its utilization of a large, longitudinal cohort comprising ANCA vasculitis patients who diligently completed surveys over their disease course. Furthermore, the study’s reliance on patient-reported data directly extracted from surveys offers a genuine reflection of the patients’ experiences, devoid of clinician interpretations. The intentionally broad scope of the surveys was designed to accommodate patient-identified exposures. However, this broad approach, coupled with the utilization of open-ended questions, imposes limitations on the systematic collection of data for each stress-related event. We acknowledge that participants with clinician concern of relapse had more frequent clinical follow-up visits over an extended duration in comparison to those participants in stable remission. To mitigate these constraints, we carefully reviewed survey responses and translated the longitudinal, qualitative responses into categorical data suitable for quantitative analysis during a uniform 15-month timeline. Moving forward, enhancing the efficacy of future studies utilizing patient-reported data on these exposures necessitates the implementation of more comprehensive questionnaires tailored to each specific exposure of interest. Additionally, incorporating inquiries that gauge the extent of influence on patients’ day-to-day lives would further fortify the study’s findings.

While the statistical significance was not reached for any measured external exposures and stress (p=0.065 and p=0.12, respectively), we emphasize further investigation with more detailed exposure information is needed. Specifically, utilizing validated questionnaires tailored to each exposure could provide valuable insights. For example, a more nuanced questionnaire could encompass patient-reported levels of perceived stress and coping mechanisms, along with an evaluation of disease and overall health impact done in parallel. This approach would offer a more precise understanding of the interplay between psychological events and disease activity, warranting deeper exploration.

The comparable occurrence of insect bites observed in both remission and relapse cohorts suggests a limited impact on disease progression, though further investigation is warranted. Future studies of insect bites would benefit from the use of a validated questionnaire and potentially adopting weekly or monthly surveys to better capture episodic exposure and enhance recall (11, 12). Examining the frequency of insect bites pre-diagnosis, alongside during the disease course, could offer a more comprehensive understanding of environmental vector risk factors in ANCA vasculitis.

This study offers valuable insights into the influence of patient-reported stressors and their effects on the course of ANCA vasculitis. While current literature primarily relies on medical record chart abstraction and clinician interpretation, our study underscores the significance of understanding disease events from the perspectives of patients. The inclusion of patient-reported survey responses throughout the disease course provides additional and unique data to assess the risk of disease flare in ANCA vasculitis from the perception and experience of the patient.

## Conclusion

Increased frequency of patient-reported upper respiratory infections may heighten vulnerability to relapse, even when occurring as far as 15 months prior to relapse. Providing counseling and vigilant monitoring of patients following infectious symptoms could facilitate earlier detection of disease flares. Deeper investigation into stressors and effective coping strategies is essential for elucidating their connection with relapse. Notably, our study found no significant correlation between insect bites and relapse risk.

## Data availability statement

The original contributions presented in the study are included in the article/**Supplementary Material**. Further inquiries can be directed to the corresponding author.

## Ethics statement

The studies involving humans were approved by the Office of Human Research Ethics (OHRE) and UNC Institutional Review Board. The studies were conducted in accordance with the local legislation and institutional requirements. Written informed consent for participation in this study was provided by the participant or the participants’ legal guardians/next of kin.

## Author contributions

MMC: Writing – original draft, Writing – review & editing. DPC: Writing – review & editing, Writing – original draft. YH: Writing – review & editing, Writing – original draft, Formal analysis. LNB: Writing – review & editing. VKD: Writing – review & editing. EYW: Writing – review & editing. KJ: Writing – review & editing. NO: Writing – review & editing. CJP: Writing – review & editing. CDH: Writing – review & editing. RJF: Writing – review & editing. SLH: Writing – review & editing, Writing – original draft, Formal analysis.
