# Supplementary material for: Examining the role of patient-reported external factors and risk of relapse in anti-neutrophilic cytoplasmic autoantibody vasculitis
Source: Front Nephrol. 2024 Jul 2;4:1404451. doi: 10.3389/fneph.2024.1404451 (PMC11249541; doi:10.3389/fneph.2024.1404451)

Supplementary Material

Examining the role of patient-reported external factors with risk of anti-neutrophilic cytoplasmic autoantibody (ANCA) vasculitis relapse

Mary M. Collie, Dhruti P. Chen*, Yichun Hu, Lauren N. Blazek, Vimal K. Derebail, Eveline Y. Wu, Koyal Jain, Nicole Orzechowski, Caroline J. Poulton, Candace D. Henderson, Ronald J. Falk, and Susan L. Hogan

*** Correspondence:** Dhruti P. Chen: dhruti_chen@med.unc.edu

# Supplementary Methods

## Definition or Formula

The Rate of Average Infection Events in three months for infection type

=$\frac{Average of Infection Event}{Total Observation Time for Infection Type in 3 months}$

$$= \frac{\sum Numbers of Infection Events}{\sum Survey Numbers \times\sum Persons of a type in 3 months}$$

The formula’s unit of rate of average infection events is the event number per survey and person in three months. The unit of the three months rate of average event with an integer is used in this study by event numbers for per 100 survey and 100 patients in three months.

# Supplementary Tables

**Supplementary Table 2-1.** Characterization of exposures for relapse and remission in the PR3 serotype subgroup

| Variables: n (%) or Median (IQR) | Relapse Cohort  N=37 | Remission Cohort  N=19 | P value * |
| --- | --- | --- | --- |
| Survey Number | 138 | 67 |  |
| Surveys per Subject | 23(15,26) | 8(6,13) | <0.0001 |
| Total External Exposures | 36(97%) | 18(95%) | 1.00 |
|  |  |  |  |
| Any Stressors | 32(87%) | 15(79%) | 0.47 |
| Number of Stressors | 3(1,3) | 2(1,3) | 0.24 |
| Insect Bites | 12(32%) | 8(42%) | 0.56 |
| Number of Insect Bites | 0(0,1) | 0(0,1) | 0.42 |
| Infections | 27(73%) | 12(63%) | 0.11 |
| Number of Infections | 1(0,2) | 1(0,2) | 0.33 |

*P values were calculated by Fisher Exact Test for categorical variables and Wilcoxon Two Sample Test for continuous variables

**Supplementary Table 2-2.** Characterization of exposures for relapse and remission in the MPO serotype subgroup

| Variables: n (%) or Median (IQR) | Relapse Cohort  N = 27 | Remission Cohort  N = 24 | P value * |
| --- | --- | --- | --- |
| Survey Number | 100 | 66 |  |
| Surveys per Subject | 15(13,24) | 5(3,10) | <0.0001 |
| Total External Exposures | 26(96%) | 20(83%) | 0.17 |
|  |  |  |  |
| Any Stressors | 25(93%) | 19(79%) | 0.23 |
| Number of Stressors | 1(1,3) | 2(1,3) | 0.76 |
| Insect Bites | 9(33%) | 5(25%) | 0.55 |
| Number of Insect Bites | 0(0,1) | 0(0,1) | 0.45 |
| Infections | 18(67%) | 12(50%) | 0.27 |
| Number of Infections | 1(0,2) | 1(0,2) | 0.11 |

*P values were calculated by Fisher Exact Test for categorical variables and Wilcoxon Two Sample Test for continuous variables

# Supplementary Figures

## Supplementary Figure 3-1. Relapse and remission timeline for survey review

##
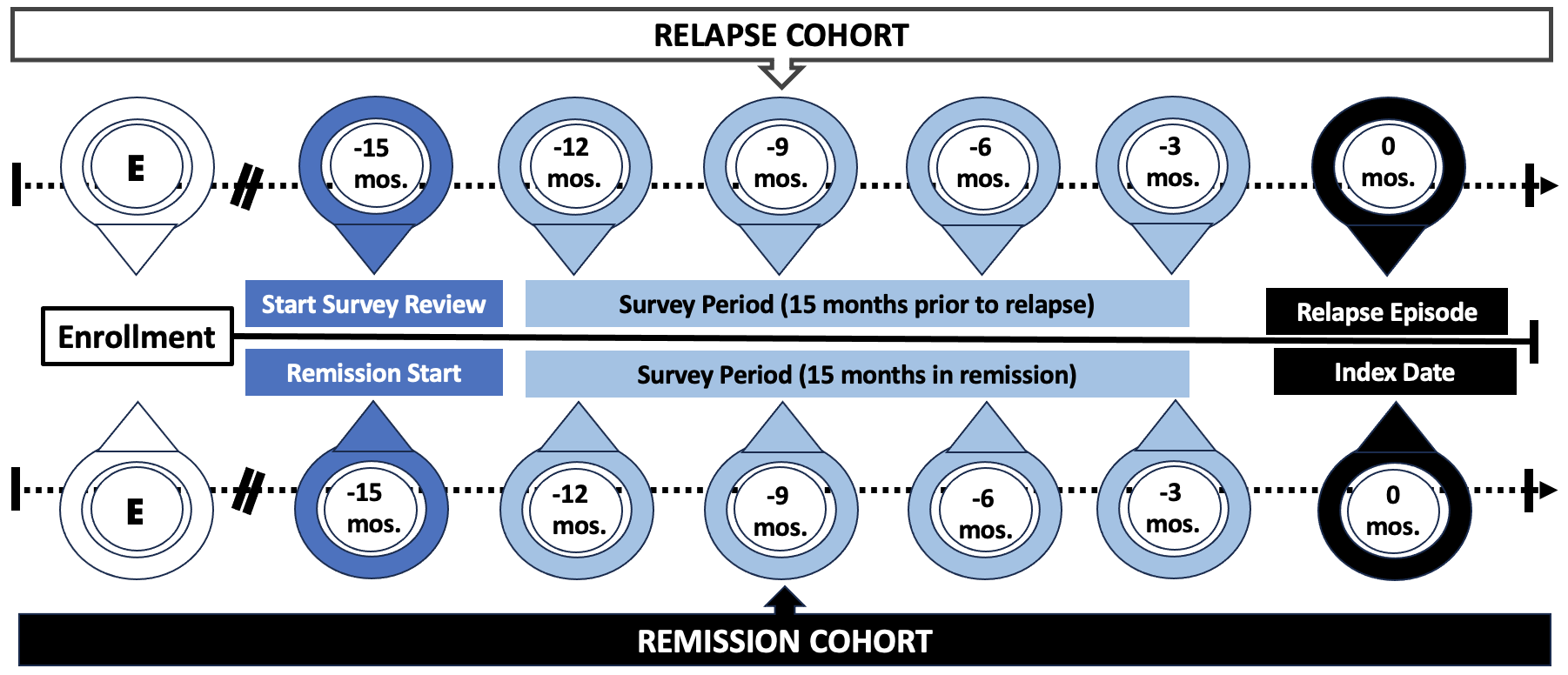


**Supplementary Figure 3-2A - D.** Infection subtypes as reported by patients prior to index date of remission or relapse. The average infection rates are reported using per 100 persons with 100 measures within three months. P values were reported using the Exact Cochran-Armitage Test.


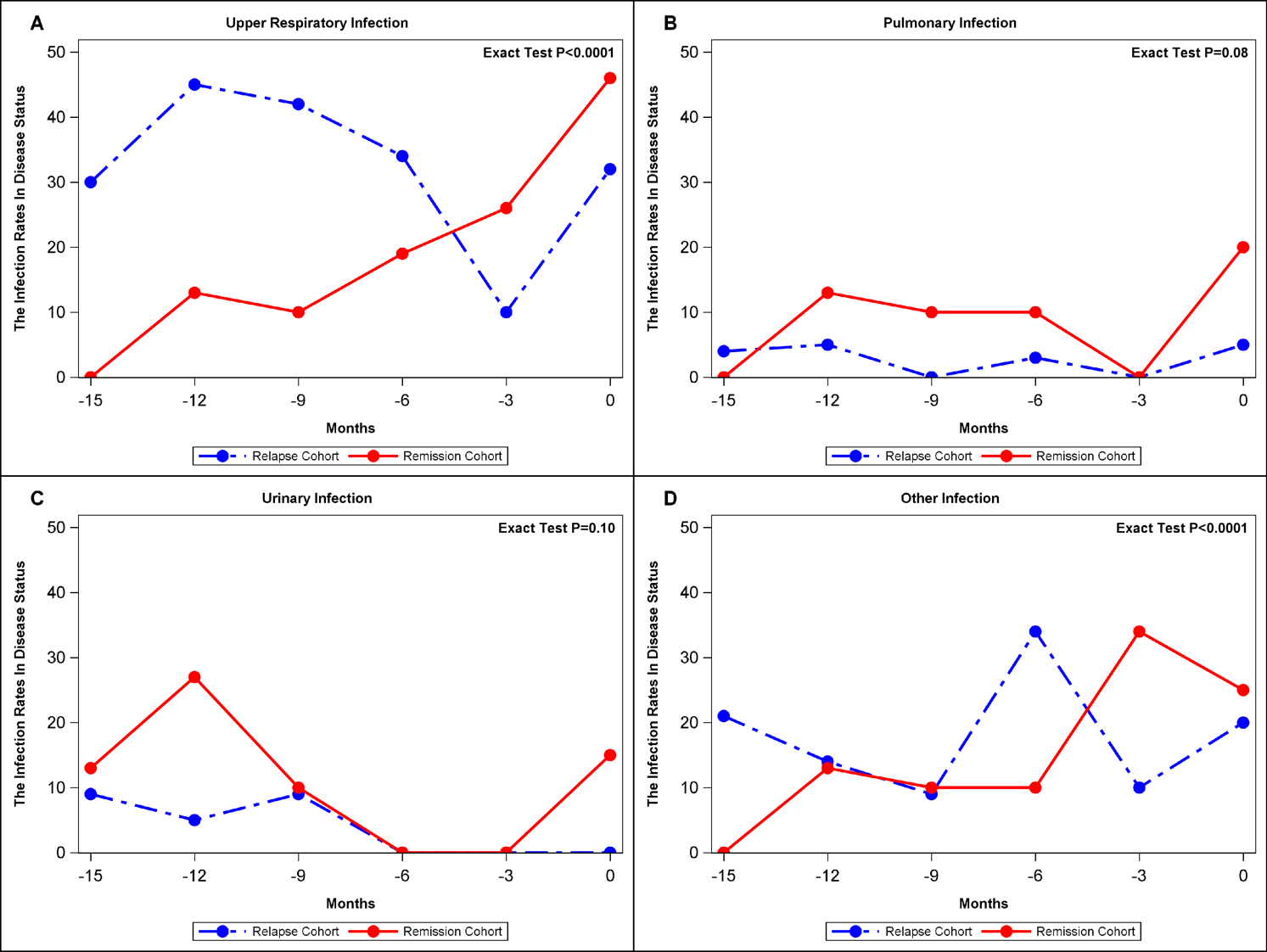

Supplement: Supplementary file 1 [file DataSheet_1.docx]
